# 1-Benzyl-2-phenyl-1*H*-benzimidazole–4,4′-(cyclo­hexane-1,1-di­yl)diphenol (1/1)

**DOI:** 10.1107/S1600536811024007

**Published:** 2011-06-25

**Authors:** Chunhua Ge, Rui Zhang, Xiangdong Zhang, Su Li

**Affiliations:** a College of Chemistry, Liaoning University, Shenyang, Liaoning 110036, People’s Republic of China

## Abstract

The asymmetric unit of the title co-crystal, C_20_H_16_N_2_·C_18_H_20_O_2_, contains one mol­ecule of 4,4′-(cyclo­hexane-1,1-di­yl)diphenol (in which the cyclo­hexane ring adopts a chair conformation) and one mol­ecule of 1-benzyl-2-phenyl-1*H*-benzimidazole, which are paired through an O—H⋯N hydrogen bond. These pairs are further linked by inter­molecular O—H⋯O hydrogen bonds into chains along [010]. Weak inter­molecular C—H⋯O and C—H⋯π inter­actions further consolidate the crystal packing. The dihedral angles between the pendant phenyl rings and the benzimidazole ring are 86.9 (2) and 43.1 (2)°.

## Related literature

For the synthesis of 1,1-bis­(4-hy­droxy­phen­yl)cyclo­hexane, see: Yoshizawa *et al.*(2007 ▶). For related structures, see: Caira *et al.* (1995 ▶, 1997 ▶); Coupar *et al.* (1997 ▶); Lavy & Kaftory (2006 ▶); MacLean *et al.* (1999 ▶).
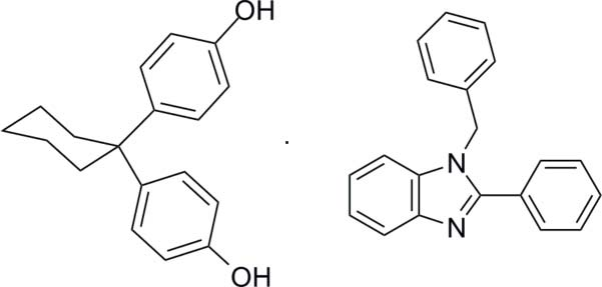


## Experimental

### 

#### Crystal data

C_20_H_16_N_2_·C_18_H_20_O_2_
*M* *_r_* = 552.69
Triclinic, 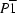

*a* = 10.448 (3) Å
*b* = 10.853 (3) Å
*c* = 14.462 (4) Å
α = 102.518 (5)°
β = 94.156 (5)°
γ = 108.605 (5)°
*V* = 1499.5 (7) Å^3^
*Z* = 2
Mo *K*α radiation
μ = 0.08 mm^−1^
*T* = 293 K
0.20 × 0.18 × 0.18 mm

#### Data collection

Bruker SMART CCD area-detector diffractometer
Absorption correction: multi-scan (*SADABS*; Bruker, 2001 ▶) *T*_min_ = 0.982, *T*_max_ = 0.989
8312 measured reflections
5714 independent reflections
3748 reflections with *I* > 2σ(*I*)
*R*_int_ = 0.023

#### Refinement

*R*[*F* ^2^ > 2σ(*F* ^2^)] = 0.069
*wR*(*F* ^2^) = 0.140
*S* = 1.07
5714 reflections
381 parameters
H-atom parameters constrained
Δρ_max_ = 0.19 e Å^−3^
Δρ_min_ = −0.20 e Å^−3^

### 

Data collection: *SMART* (Bruker, 2001 ▶); cell refinement: *SAINT* (Bruker, 2001 ▶); data reduction: *SAINT*; program(s) used to solve structure: *SHELXS97* (Sheldrick, 2008 ▶); program(s) used to refine structure: *SHELXL97* (Sheldrick, 2008 ▶); molecular graphics: *SHELXTL* (Sheldrick, 2008 ▶); software used to prepare material for publication: *SHELXL97*, *PLATON* (Spek, 2009 ▶) and *WinGX* (Farrugia, 1999 ▶).

## Supplementary Material

Crystal structure: contains datablock(s) I, global. DOI: 10.1107/S1600536811024007/cv5112sup1.cif

Structure factors: contains datablock(s) I. DOI: 10.1107/S1600536811024007/cv5112Isup2.hkl

Supplementary material file. DOI: 10.1107/S1600536811024007/cv5112Isup3.cml

Additional supplementary materials:  crystallographic information; 3D view; checkCIF report

## Figures and Tables

**Table 1:** Hydrogen-bond geometry (Å, °) *Cg* is the centroid of the C19–C24 ring.

*D*—H⋯*A*	*D*—H	H⋯*A*	*D*⋯*A*	*D*—H⋯*A*
O1—H1⋯N2	0.82	1.87	2.677 (3)	166
O2—H2⋯O1^i^	0.82	1.91	2.718 (3)	168
C29—H29⋯O2^ii^	0.93	2.64	3.467 (4)	148
C32—H32*A*⋯*Cg*^iii^	0.97	2.77	3.403 (4)	123

Symmetry codes: (i) 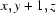; (ii) 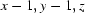; (iii) 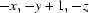.

**Fig. 1.**
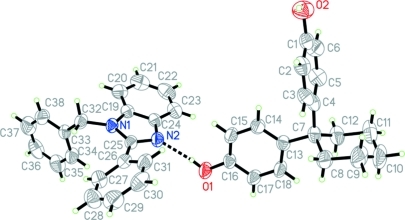
A content of asymmetric unit of (I), with displacement ellipsoids shown at the 50% probability level. Dashed line denotes hydrogen bond.

**Fig. 2.**
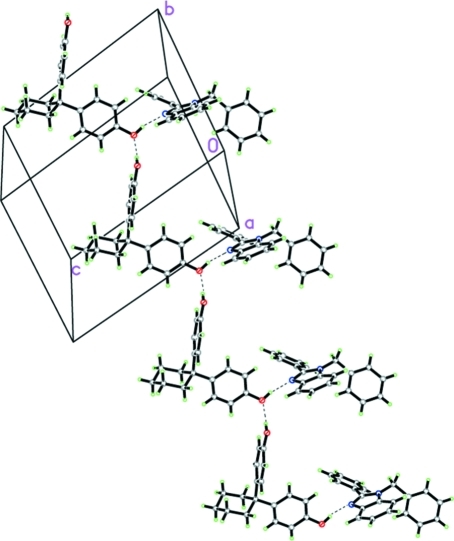
A portion of the crystal packing showing hydrogen-bonded (dashed lines) chains.

## supplementary crystallographic information

### Comment

1,1-Bis(4-hydroxyphenyl)cyclohexane(BHC) is one of the most popular candidate for efficient and versatile synthesis of self-organized systems with specific properties and functions. The groups of BHC can participate in intermolecular hydrogen bonding, π···π and other interactions(for examples, see: Caira *et al.*, 1995,1997; Coupar *et al.*,1997; Lavy & Kaftory, 2006; MacLean *et al.*, 1999). BHC forms not only inclusion complex with neutral molecule but also supramolecular framework with the organic base. Here, we report the 1:1 cocrystal of BHC with 1-benzyl-2-phenyl-1*H*-benzo[*d*]imidazole(BPBI), a *N*-containing compound.

The asymmetric unit of the title cocrystal (Fig. 1) contains one molecule of BHC and one molecule of 1-benzyl-2-phenyl-1*H*-benzo[*d*]imidazole, which are paired through the O—H···N hydrogen bond (Table 1). These pairs are further linked by intermolecular O—H···O hydrogen bonds (Table 1) into chains in [010] (Fig. 2). Weak intermolecular C—H···O and C—H···π interactions (Table 1) consolidate further the crystal packing.

### Experimental

1,1-Bis(4-hydroxyphenyl)cyclohexane (BHC) was synthesized according to the known procedure (Yoshizawa *et al.*, 2007). To the BHC (0.1 mmol) in methanol (25 ml) was added 1-benzyl-2-phenyl-1*H*-benzo[*d*]imidazole (0.1 mmol) in methanol(5 ml) dropwise. The resulting mixture was stirred for 2 h at room temperature then filtered. Crystals suitable for X-ray analysis were obtained by slow evaporation of the solvent in a few days.

### Refinement

H atoms were positioned geometrically (C—H 0.93-0.97 Å; O—H 0.82 Å), and refined using a riding model, with *U*_iso_(H) = 1.2-1.5 *U*_eq_(C, O).

### Crystal data

**Table:**

C_20_H_16_N_2_·C_18_H_20_O_2_	*Z* = 2
*M**_r_* = 552.69	*F*(000) = 588
Triclinic, *P*1	*D*_x_ = 1.224 Mg m^−^^3^
Hall symbol: -P 1	Mo *K*α radiation, λ = 0.71073 Å
*a* = 10.448 (3) Å	Cell parameters from 184 reflections
*b* = 10.853 (3) Å	θ = 2.5–22.6°
*c* = 14.462 (4) Å	µ = 0.08 mm^−^^1^
α = 102.518 (5)°	*T* = 293 K
β = 94.156 (5)°	Block, colourless
γ = 108.605 (5)°	0.20 × 0.18 × 0.18 mm
*V* = 1499.5 (7) Å^3^	

### Data collection

**Table:**

Bruker SMART CCD area-detector diffractometer	5714 independent reflections
Radiation source: fine-focus sealed tube	3748 reflections with *I* > 2σ(*I*)
graphite	*R*_int_ = 0.023
φ and ω scans	θ_max_ = 26.0°, θ_min_ = 1.5°
Absorption correction: multi-scan (*SADABS*; Bruker, 2001)	*h* = −12→6
*T*_min_ = 0.982, *T*_max_ = 0.989	*k* = −13→13
8312 measured reflections	*l* = −16→17

### Refinement

**Table:**

Refinement on *F*^2^	Primary atom site location: structure-invariant direct methods
Least-squares matrix: full	Secondary atom site location: difference Fourier map
*R*[*F*^2^ > 2σ(*F*^2^)] = 0.069	Hydrogen site location: inferred from neighbouring sites
*wR*(*F*^2^) = 0.140	H-atom parameters constrained
*S* = 1.07	*w* = 1/[σ^2^(*F*_o_^2^) + (0.0378*P*)^2^ + 0.5518*P*] where *P* = (*F*_o_^2^ + 2*F*_c_^2^)/3
5714 reflections	(Δ/σ)_max_ < 0.001
381 parameters	Δρ_max_ = 0.19 e Å^−^^3^
0 restraints	Δρ_min_ = −0.20 e Å^−^^3^

### Special details

**Table:**

Geometry. All e.s.d.'s (except the e.s.d. in the dihedral angle between two l.s. planes) are estimated using the full covariance matrix. The cell e.s.d.'s are taken into account individually in the estimation of e.s.d.'s in distances, angles and torsion angles; correlations between e.s.d.'s in cell parameters are only used when they are defined by crystal symmetry. An approximate (isotropic) treatment of cell e.s.d.'s is used for estimating e.s.d.'s involving l.s. planes.
Refinement. Refinement of *F*^2^ against ALL reflections. The weighted *R*-factor *wR* and goodness of fit *S* are based on *F*^2^, conventional *R*-factors *R* are based on *F*, with *F* set to zero for negative *F*^2^. The threshold expression of *F*^2^ > σ(*F*^2^) is used only for calculating *R*-factors(gt) *etc*. and is not relevant to the choice of reflections for refinement. *R*-factors based on *F*^2^ are statistically about twice as large as those based on *F*, and *R*- factors based on ALL data will be even larger.

### Fractional atomic coordinates and isotropic or equivalent isotropic displacement parameters (Å^2^)

**Table:**

	*x*	*y*	*z*	*U*_iso_*/*U*_eq_	
N2	0.0175 (2)	0.4318 (2)	0.19242 (13)	0.0456 (5)	
O1	0.1024 (2)	0.47607 (17)	0.37964 (12)	0.0580 (5)	
H1	0.0730	0.4737	0.3249	0.087*	
C4	0.2918 (2)	1.1141 (2)	0.57332 (18)	0.0468 (6)	
C16	0.1443 (2)	0.6055 (2)	0.43496 (17)	0.0445 (6)	
C13	0.2359 (2)	0.8657 (2)	0.55696 (17)	0.0439 (6)	
O2	0.3146 (2)	1.3869 (2)	0.40445 (16)	0.0745 (6)	
H2	0.2436	1.4035	0.3989	0.112*	
C7	0.2832 (2)	1.0050 (3)	0.62764 (17)	0.0480 (6)	
C2	0.1855 (3)	1.2431 (3)	0.49660 (19)	0.0557 (7)	
H2A	0.1089	1.2661	0.4837	0.067*	
C18	0.2299 (3)	0.7534 (3)	0.59002 (18)	0.0566 (7)	
H18	0.2573	0.7651	0.6549	0.068*	
C14	0.1943 (3)	0.8422 (3)	0.45984 (18)	0.0501 (7)	
H14	0.1971	0.9145	0.4344	0.060*	
C15	0.1486 (3)	0.7141 (3)	0.39961 (18)	0.0531 (7)	
H15	0.1205	0.7015	0.3347	0.064*	
C1	0.3033 (3)	1.2990 (3)	0.46111 (18)	0.0524 (7)	
C5	0.4091 (3)	1.1736 (3)	0.5369 (2)	0.0600 (8)	
H5	0.4861	1.1509	0.5494	0.072*	
C17	0.1853 (3)	0.6261 (3)	0.53100 (18)	0.0551 (7)	
H17	0.1829	0.5535	0.5561	0.066*	
C3	0.1813 (3)	1.1523 (3)	0.55157 (19)	0.0532 (7)	
H3	0.1007	1.1156	0.5748	0.064*	
C8	0.1806 (3)	1.0029 (3)	0.69993 (19)	0.0614 (8)	
H8A	0.0931	0.9937	0.6662	0.074*	
H8B	0.1678	0.9245	0.7250	0.074*	
C12	0.4235 (3)	1.0307 (3)	0.6858 (2)	0.0631 (8)	
H12A	0.4185	0.9541	0.7115	0.076*	
H12B	0.4917	1.0389	0.6433	0.076*	
C6	0.4154 (3)	1.2649 (3)	0.4827 (2)	0.0644 (8)	
H6	0.4964	1.3037	0.4606	0.077*	
C9	0.2249 (4)	1.1275 (3)	0.7835 (2)	0.0811 (10)	
H9A	0.2286	1.2054	0.7599	0.097*	
H9B	0.1584	1.1172	0.8273	0.097*	
C11	0.4678 (3)	1.1575 (3)	0.7681 (2)	0.0827 (10)	
H11A	0.5558	1.1692	0.8025	0.099*	
H11B	0.4779	1.2351	0.7425	0.099*	
C10	0.3643 (4)	1.1491 (4)	0.8364 (2)	0.0944 (12)	
H10A	0.3933	1.2316	0.8871	0.113*	
H10B	0.3586	1.0753	0.8655	0.113*	
N1	−0.07922 (19)	0.33432 (19)	0.04038 (13)	0.0413 (5)	
C25	−0.0994 (2)	0.3712 (2)	0.13303 (16)	0.0404 (6)	
C33	−0.1767 (2)	0.1317 (2)	−0.09418 (17)	0.0426 (6)	
C24	0.1194 (2)	0.4352 (2)	0.13605 (18)	0.0432 (6)	
C19	0.0607 (3)	0.3762 (2)	0.04057 (17)	0.0424 (6)	
C31	−0.2442 (3)	0.4599 (3)	0.23510 (17)	0.0543 (7)	
H31	−0.1693	0.5385	0.2583	0.065*	
C32	−0.1785 (3)	0.2693 (2)	−0.04718 (17)	0.0483 (6)	
H32A	−0.1588	0.3253	−0.0920	0.058*	
H32B	−0.2692	0.2614	−0.0320	0.058*	
C26	−0.2322 (2)	0.3557 (3)	0.16609 (17)	0.0454 (6)	
C27	−0.3451 (3)	0.2390 (3)	0.1330 (2)	0.0581 (7)	
H27	−0.3386	0.1673	0.0873	0.070*	
C34	−0.1608 (3)	0.0442 (3)	−0.04193 (19)	0.0553 (7)	
H34	−0.1518	0.0694	0.0246	0.066*	
C20	0.1377 (3)	0.3717 (3)	−0.0330 (2)	0.0561 (7)	
H20	0.0974	0.3337	−0.0967	0.067*	
C38	−0.1905 (3)	0.0911 (3)	−0.19258 (19)	0.0587 (7)	
H38	−0.2022	0.1483	−0.2293	0.070*	
C23	0.2607 (3)	0.4865 (3)	0.1614 (2)	0.0598 (8)	
H23	0.3016	0.5230	0.2251	0.072*	
C36	−0.1697 (3)	−0.1183 (3)	−0.1844 (3)	0.0725 (9)	
H36	−0.1657	−0.2015	−0.2148	0.087*	
C30	−0.3654 (3)	0.4488 (3)	0.2699 (2)	0.0656 (8)	
H30	−0.3721	0.5196	0.3165	0.079*	
C35	−0.1581 (3)	−0.0805 (3)	−0.0871 (2)	0.0673 (8)	
H35	−0.1483	−0.1390	−0.0510	0.081*	
C37	−0.1872 (3)	−0.0328 (3)	−0.2369 (2)	0.0742 (9)	
H37	−0.1970	−0.0589	−0.3034	0.089*	
C22	0.3371 (3)	0.4812 (3)	0.0887 (3)	0.0704 (9)	
H22	0.4319	0.5152	0.1035	0.084*	
C21	0.2769 (3)	0.4265 (3)	−0.0069 (3)	0.0708 (9)	
H21	0.3325	0.4271	−0.0543	0.085*	
C29	−0.4765 (3)	0.3332 (4)	0.2357 (2)	0.0748 (9)	
H29	−0.5586	0.3257	0.2590	0.090*	
C28	−0.4667 (3)	0.2288 (3)	0.1675 (2)	0.0713 (9)	
H28	−0.5422	0.1508	0.1443	0.086*	

### Atomic displacement parameters (Å^2^)

**Table:**

	*U*^11^	*U*^22^	*U*^33^	*U*^12^	*U*^13^	*U*^23^
N2	0.0505 (12)	0.0408 (13)	0.0384 (12)	0.0127 (10)	0.0006 (9)	0.0022 (10)
O1	0.0849 (14)	0.0406 (11)	0.0403 (10)	0.0203 (9)	−0.0058 (9)	0.0006 (9)
C4	0.0486 (15)	0.0336 (15)	0.0524 (16)	0.0164 (11)	0.0046 (12)	−0.0035 (12)
C16	0.0545 (15)	0.0367 (15)	0.0384 (14)	0.0172 (12)	0.0031 (11)	0.0006 (12)
C13	0.0491 (14)	0.0398 (15)	0.0422 (15)	0.0184 (11)	0.0063 (11)	0.0047 (12)
O2	0.0945 (16)	0.0678 (15)	0.0760 (14)	0.0394 (12)	0.0260 (13)	0.0267 (12)
C7	0.0539 (15)	0.0401 (16)	0.0459 (15)	0.0192 (12)	0.0035 (12)	−0.0005 (12)
C2	0.0561 (17)	0.0457 (17)	0.0645 (18)	0.0246 (13)	0.0048 (13)	0.0031 (15)
C18	0.085 (2)	0.0469 (18)	0.0338 (14)	0.0258 (14)	−0.0013 (13)	0.0012 (13)
C14	0.0653 (17)	0.0399 (16)	0.0464 (16)	0.0218 (12)	0.0027 (12)	0.0101 (13)
C15	0.0729 (18)	0.0469 (18)	0.0350 (14)	0.0201 (13)	−0.0010 (12)	0.0048 (13)
C1	0.0694 (19)	0.0369 (16)	0.0470 (16)	0.0201 (13)	0.0088 (13)	0.0000 (13)
C5	0.0508 (16)	0.0599 (19)	0.074 (2)	0.0265 (14)	0.0088 (14)	0.0146 (16)
C17	0.085 (2)	0.0410 (17)	0.0388 (15)	0.0244 (14)	0.0024 (13)	0.0084 (13)
C3	0.0468 (15)	0.0411 (16)	0.0688 (18)	0.0157 (12)	0.0084 (13)	0.0070 (14)
C8	0.078 (2)	0.0527 (19)	0.0530 (17)	0.0271 (15)	0.0152 (14)	0.0029 (14)
C12	0.0691 (19)	0.0502 (18)	0.0622 (19)	0.0229 (14)	−0.0080 (14)	0.0006 (15)
C6	0.0558 (18)	0.065 (2)	0.074 (2)	0.0204 (15)	0.0182 (14)	0.0173 (17)
C9	0.117 (3)	0.068 (2)	0.060 (2)	0.045 (2)	0.0215 (19)	−0.0045 (17)
C11	0.090 (2)	0.061 (2)	0.077 (2)	0.0234 (18)	−0.0250 (19)	−0.0104 (18)
C10	0.148 (4)	0.063 (2)	0.056 (2)	0.041 (2)	−0.005 (2)	−0.0190 (18)
N1	0.0518 (12)	0.0338 (12)	0.0336 (11)	0.0154 (9)	0.0019 (9)	−0.0005 (9)
C25	0.0498 (14)	0.0319 (14)	0.0365 (14)	0.0138 (11)	0.0026 (11)	0.0043 (11)
C33	0.0444 (14)	0.0387 (15)	0.0394 (14)	0.0130 (11)	0.0009 (10)	0.0031 (12)
C24	0.0519 (15)	0.0281 (14)	0.0480 (15)	0.0147 (11)	0.0070 (12)	0.0047 (12)
C19	0.0574 (16)	0.0278 (14)	0.0430 (15)	0.0188 (11)	0.0094 (11)	0.0042 (11)
C31	0.0662 (18)	0.0578 (19)	0.0378 (15)	0.0232 (14)	0.0088 (12)	0.0067 (14)
C32	0.0638 (16)	0.0423 (16)	0.0365 (14)	0.0205 (12)	−0.0012 (11)	0.0046 (12)
C26	0.0490 (14)	0.0504 (17)	0.0387 (14)	0.0187 (12)	0.0049 (11)	0.0132 (13)
C27	0.0539 (17)	0.0554 (19)	0.0602 (18)	0.0171 (14)	0.0064 (13)	0.0086 (15)
C34	0.0666 (18)	0.0477 (18)	0.0481 (16)	0.0221 (13)	−0.0040 (13)	0.0053 (14)
C20	0.074 (2)	0.0426 (17)	0.0548 (17)	0.0247 (14)	0.0210 (14)	0.0084 (14)
C38	0.0746 (19)	0.0499 (19)	0.0454 (17)	0.0181 (14)	0.0064 (13)	0.0051 (14)
C23	0.0523 (17)	0.0498 (18)	0.071 (2)	0.0139 (13)	0.0010 (14)	0.0111 (15)
C36	0.068 (2)	0.0382 (18)	0.095 (3)	0.0189 (14)	0.0056 (17)	−0.0159 (18)
C30	0.082 (2)	0.083 (2)	0.0453 (17)	0.0480 (19)	0.0192 (15)	0.0112 (16)
C35	0.071 (2)	0.0400 (18)	0.086 (2)	0.0220 (14)	−0.0039 (16)	0.0076 (17)
C37	0.088 (2)	0.059 (2)	0.0563 (19)	0.0181 (17)	0.0118 (16)	−0.0126 (17)
C22	0.0497 (17)	0.059 (2)	0.104 (3)	0.0182 (14)	0.0181 (17)	0.0216 (19)
C21	0.075 (2)	0.060 (2)	0.088 (2)	0.0309 (17)	0.0385 (18)	0.0202 (18)
C29	0.0573 (19)	0.103 (3)	0.073 (2)	0.0332 (19)	0.0190 (16)	0.030 (2)
C28	0.0513 (18)	0.076 (2)	0.080 (2)	0.0163 (15)	0.0083 (15)	0.0151 (19)

### Geometric parameters (Å, °)

**Table:**

N2—C25	1.323 (3)	C10—H10A	0.9700
N2—C24	1.383 (3)	C10—H10B	0.9700
O1—C16	1.369 (3)	N1—C25	1.365 (3)
O1—H1	0.8200	N1—C19	1.385 (3)
C4—C3	1.383 (3)	N1—C32	1.460 (3)
C4—C5	1.391 (3)	C25—C26	1.471 (3)
C4—C7	1.541 (4)	C33—C34	1.376 (4)
C16—C17	1.372 (3)	C33—C38	1.378 (3)
C16—C15	1.373 (3)	C33—C32	1.507 (3)
C13—C14	1.383 (3)	C24—C23	1.391 (3)
C13—C18	1.388 (3)	C24—C19	1.391 (3)
C13—C7	1.534 (3)	C19—C20	1.381 (3)
O2—C1	1.369 (3)	C31—C30	1.376 (4)
O2—H2	0.8200	C31—C26	1.382 (4)
C7—C12	1.547 (3)	C31—H31	0.9300
C7—C8	1.549 (3)	C32—H32A	0.9700
C2—C1	1.377 (4)	C32—H32B	0.9700
C2—C3	1.386 (4)	C26—C27	1.388 (3)
C2—H2A	0.9300	C27—C28	1.379 (4)
C18—C17	1.369 (3)	C27—H27	0.9300
C18—H18	0.9300	C34—C35	1.379 (4)
C14—C15	1.382 (3)	C34—H34	0.9300
C14—H14	0.9300	C20—C21	1.372 (4)
C15—H15	0.9300	C20—H20	0.9300
C1—C6	1.371 (4)	C38—C37	1.372 (4)
C5—C6	1.380 (4)	C38—H38	0.9300
C5—H5	0.9300	C23—C22	1.367 (4)
C17—H17	0.9300	C23—H23	0.9300
C3—H3	0.9300	C36—C35	1.362 (4)
C8—C9	1.522 (4)	C36—C37	1.364 (5)
C8—H8A	0.9700	C36—H36	0.9300
C8—H8B	0.9700	C30—C29	1.373 (4)
C12—C11	1.527 (4)	C30—H30	0.9300
C12—H12A	0.9700	C35—H35	0.9300
C12—H12B	0.9700	C37—H37	0.9300
C6—H6	0.9300	C22—C21	1.393 (4)
C9—C10	1.518 (5)	C22—H22	0.9300
C9—H9A	0.9700	C21—H21	0.9300
C9—H9B	0.9700	C29—C28	1.368 (4)
C11—C10	1.511 (5)	C29—H29	0.9300
C11—H11A	0.9700	C28—H28	0.9300
C11—H11B	0.9700		
			
C25—N2—C24	105.9 (2)	C9—C10—H10A	109.6
C16—O1—H1	109.5	C11—C10—H10B	109.6
C3—C4—C5	115.6 (3)	C9—C10—H10B	109.6
C3—C4—C7	122.6 (2)	H10A—C10—H10B	108.1
C5—C4—C7	121.7 (2)	C25—N1—C19	107.00 (18)
O1—C16—C17	117.2 (2)	C25—N1—C32	129.9 (2)
O1—C16—C15	123.8 (2)	C19—N1—C32	123.0 (2)
C17—C16—C15	118.9 (2)	N2—C25—N1	111.9 (2)
C14—C13—C18	116.0 (2)	N2—C25—C26	121.9 (2)
C14—C13—C7	124.1 (2)	N1—C25—C26	126.0 (2)
C18—C13—C7	119.9 (2)	C34—C33—C38	118.2 (3)
C1—O2—H2	109.5	C34—C33—C32	122.2 (2)
C13—C7—C4	110.2 (2)	C38—C33—C32	119.6 (2)
C13—C7—C12	109.1 (2)	N2—C24—C23	130.2 (2)
C4—C7—C12	110.9 (2)	N2—C24—C19	109.5 (2)
C13—C7—C8	107.9 (2)	C23—C24—C19	120.3 (2)
C4—C7—C8	111.6 (2)	C20—C19—N1	131.8 (2)
C12—C7—C8	106.9 (2)	C20—C19—C24	122.5 (2)
C1—C2—C3	120.0 (3)	N1—C19—C24	105.7 (2)
C1—C2—H2A	120.0	C30—C31—C26	120.9 (3)
C3—C2—H2A	120.0	C30—C31—H31	119.6
C17—C18—C13	122.7 (2)	C26—C31—H31	119.6
C17—C18—H18	118.7	N1—C32—C33	112.5 (2)
C13—C18—H18	118.7	N1—C32—H32A	109.1
C15—C14—C13	121.8 (2)	C33—C32—H32A	109.1
C15—C14—H14	119.1	N1—C32—H32B	109.1
C13—C14—H14	119.1	C33—C32—H32B	109.1
C16—C15—C14	120.4 (2)	H32A—C32—H32B	107.8
C16—C15—H15	119.8	C31—C26—C27	118.5 (2)
C14—C15—H15	119.8	C31—C26—C25	118.8 (2)
O2—C1—C6	117.8 (3)	C27—C26—C25	122.7 (2)
O2—C1—C2	123.5 (3)	C28—C27—C26	120.4 (3)
C6—C1—C2	118.7 (3)	C28—C27—H27	119.8
C6—C5—C4	122.4 (3)	C26—C27—H27	119.8
C6—C5—H5	118.8	C33—C34—C35	120.7 (3)
C4—C5—H5	118.8	C33—C34—H34	119.6
C18—C17—C16	120.1 (3)	C35—C34—H34	119.6
C18—C17—H17	120.0	C21—C20—C19	116.4 (3)
C16—C17—H17	120.0	C21—C20—H20	121.8
C4—C3—C2	122.8 (3)	C19—C20—H20	121.8
C4—C3—H3	118.6	C37—C38—C33	120.6 (3)
C2—C3—H3	118.6	C37—C38—H38	119.7
C9—C8—C7	114.1 (2)	C33—C38—H38	119.7
C9—C8—H8A	108.7	C22—C23—C24	117.2 (3)
C7—C8—H8A	108.7	C22—C23—H23	121.4
C9—C8—H8B	108.7	C24—C23—H23	121.4
C7—C8—H8B	108.7	C35—C36—C37	119.3 (3)
H8A—C8—H8B	107.6	C35—C36—H36	120.3
C11—C12—C7	112.4 (2)	C37—C36—H36	120.3
C11—C12—H12A	109.1	C29—C30—C31	119.9 (3)
C7—C12—H12A	109.1	C29—C30—H30	120.1
C11—C12—H12B	109.1	C31—C30—H30	120.1
C7—C12—H12B	109.1	C36—C35—C34	120.4 (3)
H12A—C12—H12B	107.8	C36—C35—H35	119.8
C1—C6—C5	120.6 (3)	C34—C35—H35	119.8
C1—C6—H6	119.7	C36—C37—C38	120.7 (3)
C5—C6—H6	119.7	C36—C37—H37	119.6
C10—C9—C8	110.7 (3)	C38—C37—H37	119.6
C10—C9—H9A	109.5	C23—C22—C21	121.9 (3)
C8—C9—H9A	109.5	C23—C22—H22	119.1
C10—C9—H9B	109.5	C21—C22—H22	119.1
C8—C9—H9B	109.5	C20—C21—C22	121.7 (3)
H9A—C9—H9B	108.1	C20—C21—H21	119.2
C10—C11—C12	111.0 (3)	C22—C21—H21	119.2
C10—C11—H11A	109.4	C28—C29—C30	120.1 (3)
C12—C11—H11A	109.4	C28—C29—H29	120.0
C10—C11—H11B	109.4	C30—C29—H29	120.0
C12—C11—H11B	109.4	C29—C28—C27	120.2 (3)
H11A—C11—H11B	108.0	C29—C28—H28	119.9
C11—C10—C9	110.3 (3)	C27—C28—H28	119.9
C11—C10—H10A	109.6		

### Hydrogen-bond geometry (Å, °)

**Table:**

Cg is the centroid of the C19–C24 ring.

**Table:**

*D*—H···*A*	*D*—H	H···*A*	*D*···*A*	*D*—H···*A*
O1—H1···N2	0.82	1.87	2.677 (3)	166
O2—H2···O1^i^	0.82	1.91	2.718 (3)	168
C29—H29···O2^ii^	0.93	2.64	3.467 (4)	148
C32—H32A···Cg^iii^	0.97	2.77	3.403 (4)	123

Symmetry codes: (i) *x*, *y*+1, *z*; (ii) *x*−1, *y*−1, *z*; (iii) −*x*, −*y*+1, −*z*.

## References

[bb1] Bruker (2001). *SMART*, *SAINT* and *SADABS* Bruker AXS Inc., Madison, Wisconsin, USA.

[bb2] Caira, M. R., Horne, A., Nassimbeni, L. R., Okuda, K. & Toda, F. (1995). *J* *Chem* *Soc* *Perkin Trans* *2*, pp. 1063–1067.

[bb3] Caira, M. R., Horne, A., Nassimbeni, L. R. & Toda, F. (1997). *J* *Mater* *Chem* **7**, 2145-2149.

[bb4] Coupar, P. I., Glidewell, C. & Ferguson, G. (1997). *Acta Cryst.* B**53**, 521–533.

[bb5] Farrugia, L. J. (1999). *J. Appl. Cryst.* **32**, 837–838.

[bb6] Lavy, T. & Kaftory, M. (2006). *Acta Cryst.* E**62**, o3977–o3978.

[bb7] MacLean, E. J., Glidewell, C., Ferguson, G., Gregson, R. M. & Lough, A. J. (1999). *Acta Cryst.* C**55**, 1867–1870.

[bb8] Sheldrick, G. M. (2008). *Acta Cryst.* A**64**, 112–122. 10.1107/S0108767307043930 18156677

[bb9] Spek, A. L. (2009). *Acta Cryst.* D**65**, 148–155. 10.1107/S090744490804362X PMC2631630 19171970

[bb10] Yoshizawa, K., Toyota, S., Toda, F., Kato, M. & Csöregh, I. (2007). *CrystEngComm*, **9**, 786–792.

